# A cost-effectiveness analysis of a universal, preventative-focused, parent and infant programme

**DOI:** 10.1186/s12913-023-10492-w

**Published:** 2024-02-08

**Authors:** Gráinne E. Crealey, Gráinne Hickey, Sinead McGilloway

**Affiliations:** 1 Clinical Costing Solutions, Belfast, BT15 4EB UK; 2 Barnardos Ireland, Christchurch Sq., Dublin 8, Dublin, D08DT63 Ireland; 3 Centre for Mental Health and Community Research, Maynooth University, Maynooth, W23 F2H6 Co. Kildare Ireland

**Keywords:** Cost-effectiveness, Early parent intervention, Universal parent support, Group-based parent training

## Abstract

**Background:**

This study assessed whether a relatively newly developed Parent and Infant (PIN) parenting support programme was cost-effective when compared to services as usual (SAU).

**Methods:**

The cost-effectiveness of the PIN programme versus SAU was assessed from an Irish health and social care perspective over a 24-month timeframe and within the context of a non-randomised, controlled before-and-after trial. In total, 163 parent-infant dyads were included in the study (86 intervention, 77 control). The primary outcome measure for the economic evaluation was the Parenting Sense of Competence Scale (PSOC).

**Results:**

The average cost of the PIN programme was €647 per dyad. The mean (SE) cost (including programme costs) was €7,027 (SE €1,345) compared to €4,811 (SE €593) in the control arm, generating a (non-significant) mean cost difference of €2,216 (bootstrap 95% CI -€665 to €5,096; *p* = 0.14). The mean incremental cost-effectiveness of the PIN service was €614 per PSOC unit gained (bootstrap 95% CI €54 to €1,481). The probability that the PIN programme was cost-effective, was 87% at a willingness-to-pay of €1,000 per one unit change in the PSOC.

**Conclusions:**

Our findings suggest that the PIN programme was cost-effective at a relatively low willingness-to-pay threshold when compared to SAU. This study addresses a significant knowledge gap in the field of early intervention by providing important real world evidence on the implementation costs and cost-effectiveness of a universal early years parenting programme. The challenges involved in assessing the cost-effectiveness of preventative interventions for very young children and their parents are also discussed.

**Trial registration:**

ISRCTN17488830 (Date of registration: 27/11/15). This trial was retrospectively registered.

**Supplementary Information:**

The online version contains supplementary material available at 10.1186/s12913-023-10492-w.

## Introduction

Inadequate care, abuse and/or neglect during infancy can undermine development and impact outcomes throughout the lifespan [1–5] while leading to increased expenditure on health, social, educational and judicial services [6–8]. Parenting support which can promote positive parenting is a growing public health and human rights priority [9]. Group-based parenting programmes delivered on a targeted basis to parents of older school going children, have been found to be effective and represent good value for money [10–15]. However, there is growing interest in supports which are delivered to all parents. Universal parenting interventions aim to support the general population, rather than targeting a specific cohort of families [16]. Given the prevalence of sub-optimal parenting, as well as the range of outcomes on which parenting can have an influence, universal approaches may be more efficient [17]. They may also have greater reach than targeted approaches due to their wider availability. Additionally, the delivery of services and supports to all families may reduce any potential stigma associated with participation [18]. However, there remain significant gaps in our understanding of the effectiveness and cost-effectiveness of parenting interventions when implemented on a universal basis [19, 20], especially in the very earliest childhood years.

Empirical studies of universally available early parenting programmes have highlighted their potential effectiveness in improving parenting knowledge, skills and attitudes [21]. Limited findings have also suggested that universal early intervention may lead to modest, but sustained improvements in parenting during early childhood (i.e. when children are 3 years old) [22], although little is known about the longer-term effects of universal supports on parenting outcomes [23, 24]. A recent study found that a multimodal intervention which included group-based parent training delivered on a universal basis in primary care settings to parents of infants aged 1–2 months, led to improvements at 14 months, in child communication skills and fine motor development, suggesting that these supports may provide a cost-efficient means of enhancing early child development [25]. However, evidence of the impact of such parenting supports on child outcomes remains mixed [20, 22, 26]. Some studies have concluded that group-based interventions delivered in the earliest years are not cost-effective [19, 27], whilst others [28] have highlighted uncertainty in the probability of cost-effectiveness of universal parenting programmes. For example, recent research demonstrated borderline cost-effectiveness of the group-based Incredible Years Infant and Toddler programmes delivered as a universal, proportionate model for parent-infant dyads, although this was linked to effects on parent health rather than any improvements in child wellbeing [26, 29].

Evaluating the cost-effectiveness of universal early parenting supports is challenging. For example, the preventative focus of universal parenting programmes (i.e. preventing the emergence of difficulties or delays in child development), as opposed to risk/disease reduction (i.e. reducing difficulties or problem behaviour in at-risk groups), may limit the possibility of detecting large effects which can be demonstrably linked to economic gains/costs savings. Moreover, previous studies [19, 27] have explored the cost-effectiveness of universal parenting interventions only within the context of short-term follow-up periods (e.g. 6–12 months post-baseline assessments), and preventative effects may take time to emerge [30]. Service providers and policy makers who wish to develop and implement parenting supports in the earliest years, must consider several factors, including the payoff between costs and expected benefits of different programme options, the impact of the programme compared to the investment required, and the time/opportunity costs incurred for skilled health and social care professions. However, significant gaps in our knowledge exist with regard to implementation and delivery costs, potential health and social care cost savings and the cost-effectiveness of universally delivered early parenting interventions [14].

This study contributes to this debate by exploring the costs and cost-effectiveness of a universal early parenting intervention called the Parent and Infant (PIN) programme. The PIN programme is a preventative, universal, multi-component intervention which targets parents’ attitudes towards their parenting role and ability to sustain responsive, sensitive parenting strategies in the early years of their child’s life. Thus, this cost-effectiveness study focused on parent outcomes, particularly, their confidence and satisfaction in their parenting role.

## Methods

**Fig. 1:**
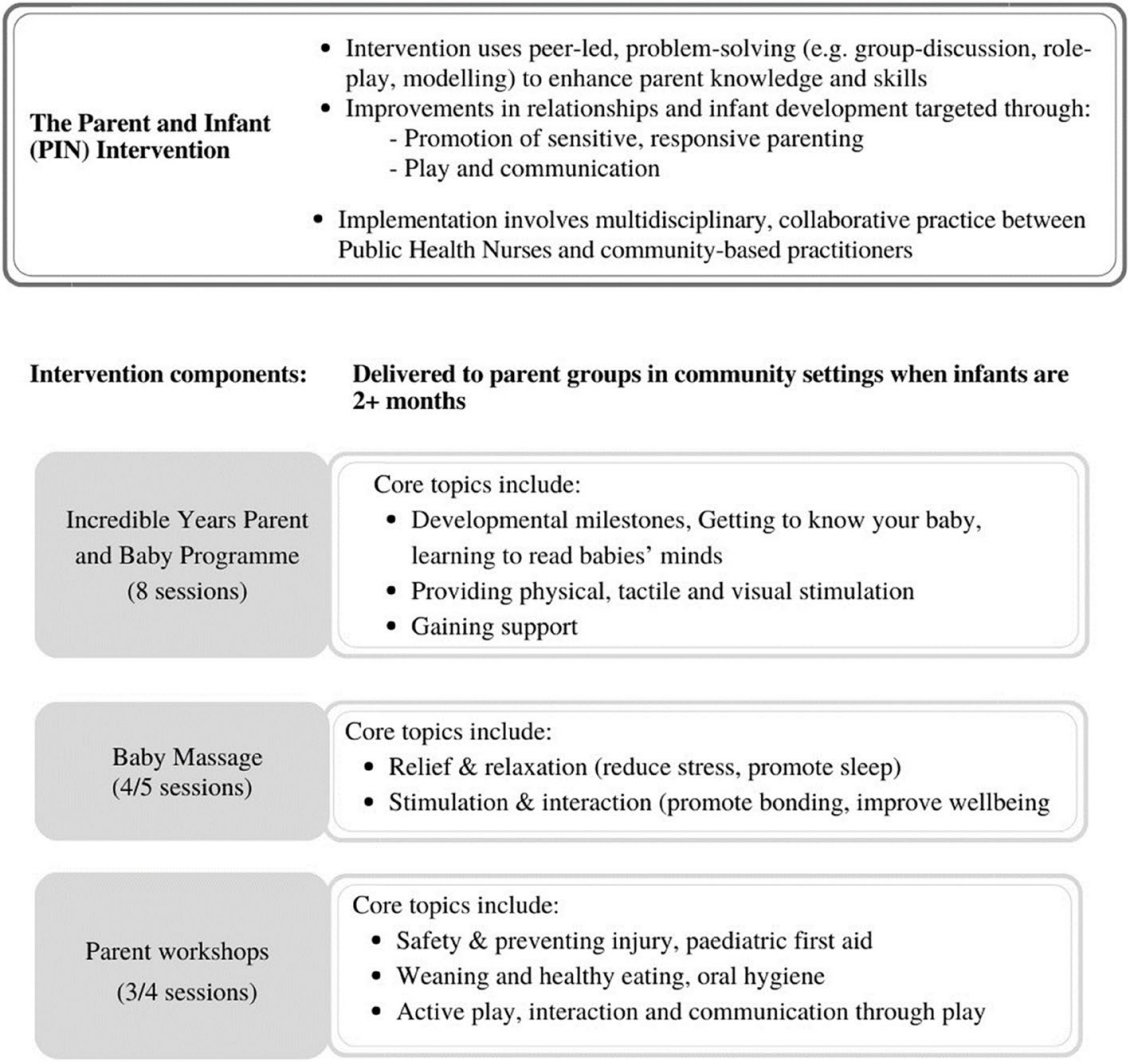
Overview of the PIN programme adapted from Hickey et al., 2020 [28]

This study considered the cost-effectiveness of the PIN programme when compared to services as usual. The PIN programme comprises 15 sessions during which parents participate in the Incredible Years Baby Programme (IYBP) and a range of complementary workshops. An overview of the PIN programme is presented in Fig. 1 with further details available on request [31].

Usual services for parent-infant dyads involve: one home visit from a Public Health Nurse (PHN) in the first 6 weeks after birth; a 2-week and 6-week check-up with a GP/hospital service; developmental check-ups with a PHN (at 3, 7 and 24 months); and free vaccinations. GP care for children under the age of eight is free in the Republic of Ireland. Breastfeeding supports and mother and baby/toddler groups are offered at a community level by public health or publicly-funded community-based services (e.g. libraries, family resource centres) and are free to access. Other services such as baby massage, baby yoga, or music classes are also available, although these are typically offered by private businesses, and parents pay to access and use them [31].

Participants were recruited to the study via public health services. Parents were eligible for inclusion if they were: (a) 16 years or older and with an infant under the age of 20 weeks; (b) willing to participate in the study; and (c) able to communicate with reasonable proficiency in English. Recruitment was conducted on a universal basis and screening for risks (e.g. socioeconomic disadvantage, early parenthood, lone parenthood) was not used as an inclusion/exclusion criterion. Once written informed consent was obtained, baseline assessments were conducted (when infants were aged 6 to 20 weeks old). Follow-up assessments took place when infants were aged approximately 8-, 16- and 24-months. Those in the intervention group were able to access the PIN programme one to three weeks after baseline assessments were completed. Parents in the intervention group also received usual services.

**Fig. 2:**
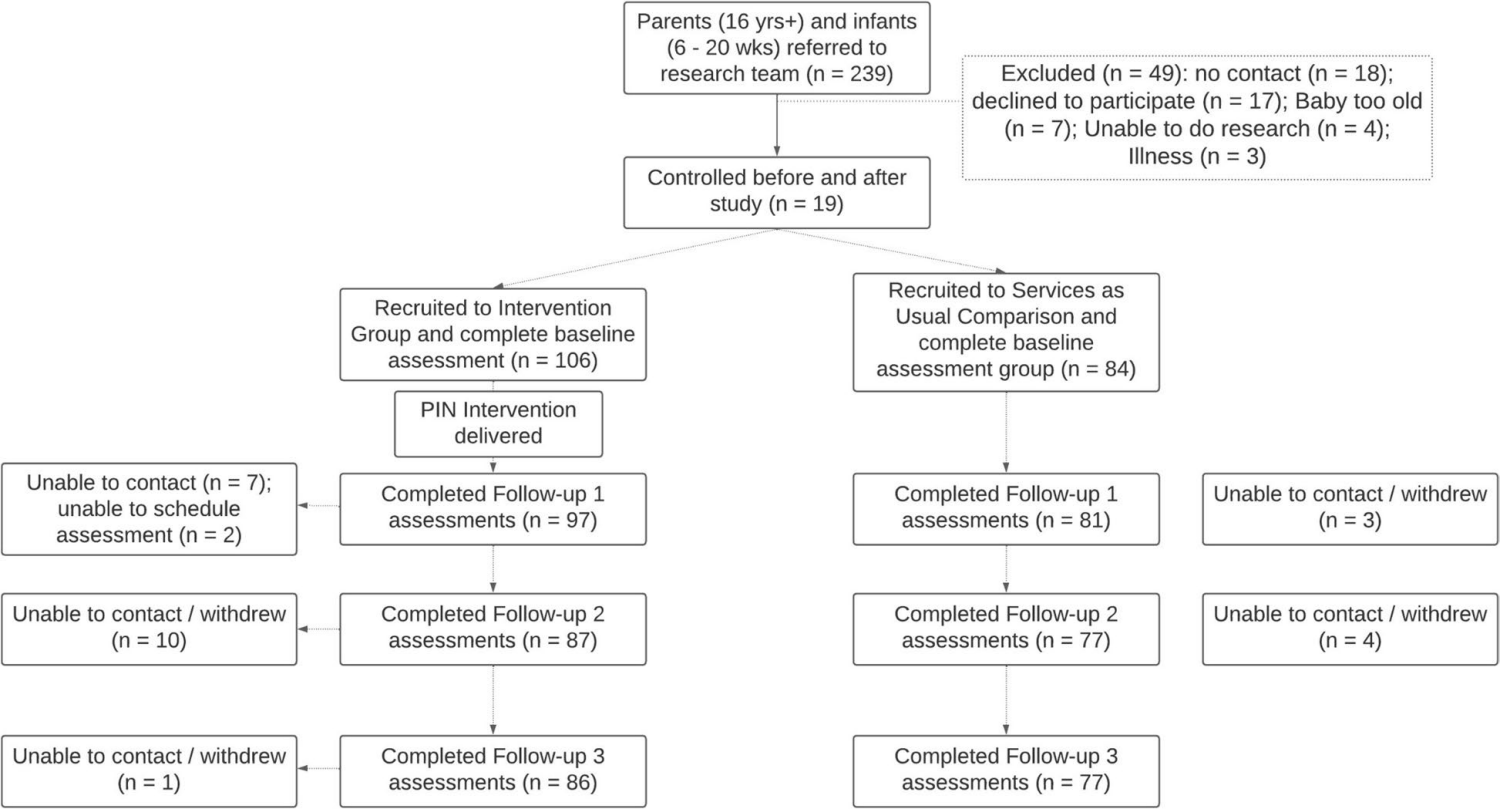
Flow of participants through the trial adapted from Hickey et al., 2020 [28]

The effectiveness of the PIN programme was assessed by means of a community-based pragmatic trial using a non-randomised, quasi-experimental, controlled before-and-after study design. The cost-effectiveness analysis was undertaken alongside this trial. The perspective for the analysis was that of the Irish health and social care system over a 24-month time frame. In total, 380 parents and infants were recruited to the trial: 106 parent-infant dyads to the PIN programme and 84 dyads to services as usual (SAU) (Fig. 2).

### Resource use and costs

A strategy was developed to estimate the incremental costs associated with the PIN programme compared to SAU involving the estimation of costs associated with the delivery of the programme and of wider health and social care resource use. Broader societal resource inputs (i.e. lost productivity) were not considered.

### Costing the PIN programme

PIN services were provided in two sites across two counties in the East and North East of the Republic of Ireland. The programme was delivered collaboratively and involved a mix of public sector and voluntary sector community-based organisations. A focus of the economic evaluation was the assessment of the cost of delivering the PIN programme in a community setting, including the cost of programme development, training of facilitators and other related staff costs of delivering group sessions, participant monitoring activities and any follow-up/management. Other related resource use was captured prospectively using activity logs completed by programme facilitators. Staff logged time related to programme preparation and delivery, indirect administrative activities, home visits and telephone contacts, as well as PIN-related training and supervision activities. The log also captured mode, distance and time spent travelling by staff and additional expenditure such as refreshments and crèche care. Costs of venue hire were captured if applicable. Where elements of the programme were delivered by independent contractors (e.g. baby massage, return to work, weaning and paediatric first aid workshops), costs charged by that organisation were was captured. With respect to the IYBP, cost estimation was based on activity logs completed by 36 facilitators across a range of sites. The total cost of delivering the PIN programme across the two sites was used to estimate an average cost per dyad.

### Collection of broader resource use data

Parental and infant/child health and social care-related resource use data were collected via a Service Utilisation Questionnaire (SUQ) based on the Client Services Receipt Inventory [32]. Data were collected on healthcare professionals’ time input (GP, nurse, health visitor, social worker, psychiatric nurse, community paediatrician), counselling and mediation services used by parents, and use of hospital services (casualty, outpatient, day procedures and overnight stays). The SUQ has been used in previous research in an Irish context to explore the cost-effectiveness of group-based parenting training programmes [13].

### Valuation of resource use

Resource use was valued by applying unit costs (e.g. the cost of health care professionals’ time, cost of an A&E visit, etc.). Unit costs were constructed based on national guidance from the Irish Health Information and Quality Authority (HIQA) on resource valuation [33]. Costs reflect Irish unit costs at 2018–19 prices and were inflated where necessary (Table 1 & Supplementary Material – Appendix 1). Total costs were aggregated for each primary caregiver, child and parent–child dyad by multiplying the quantity of each resource item over the trial period by their respective unit costs and summating.

**Table 1:** Unit costs data for service use

Service	Unit cost	Source
GP^a^	€ 52.50	ESRI publication (universal GP cost in Ireland: potential cost implications)
Nurse	€ 32.75	HSE consolidated salary scales; adjusted for Employers PRSI, pension contributions and overheads
Health visitor	€ 44.21	HSE consolidated salary scales; adjusted for Employers PRSI, pension contributions and overheads
Social Worker	€ 37.98	HSE consolidated salary scales; adjusted for Employers PRSI, pension contributions and overheads
Psychiatric nurse	€ 32.87	HSE consolidated salary scales; adjusted for Employers PRSI, pension contributions and overheads
Mediation	€ 37.57	Mediation institute of Ireland; adjusted for Employers PRSI, pension contributions and overheads
Counselling	€ 60.91	Mediation institute of Ireland; adjusted for Employers PRSI, pension contributions and overheads
A&E visit ~	€ 290.00	Healthcare Pricing Office (HPO)
Outpatient consultant appointment^b^	€ 171.00	Healthcare Pricing Office (HPO)
In-patient stay (per night)	€ 897.00	Healthcare Pricing Office (HPO)
Community paediatrician	€ 36.07	*from* O’Neill et al. (2013) adjust for inflation, employers PRSI, pension contributions and overheads
Ambulance journey**	€ 89.20	*from* O’Neill et al. (2013) adjust for inflation, employers PRSI, pension contributions and overheads

^a^https://publicpolicy.ie/digest/universal-gp-care-in-ireland-potential-cost-implications/Connelly et al. 2018. Accessed on 20/12/2023.

^b^Includes specialist appointment and other

^**^Updated based on CCEMG-EPPI-Centre Cost Converter https://eppi.ioe.ac.uk/costconversion/

### Measurement of effectiveness

The primary outcome used in the cost-effectiveness analysis was parenting sense of competence. Parenting self-efficacy is associated with several important outcomes, including lower parenting stress, better parental mental health and more responsive and sensitive parenting, as well as positive child adjustment [34–42].

A number of validated instruments exist which measure this outcome [43, 44]. The Parenting Sense of Competence (PSOC) scale [45] was chosen here because parent self-efficacy has been identified as an important mechanism for the effectiveness of early childhood interventions [46], particularly in the context of prevention-focused interventions with parents [47]. Post-intervention improvement in parenting self-efficacy is linked to reductions in parental stress, positive changes in parenting skills and long run improvements in child cognitive and social-emotional outcomes [48, 49].

The PSOC scale is a well-established 16-item self-report measure of parental competence which has been used to assess other targeted and universal parenting programmes [26, 50, 51]. The measure assesses parent anxiety, motivation and frustration, as well as perceptions of competence, capability and problem-solving in respect of the parenting role. All 16 questions are in a Likert-scale format ranging from ‘strongly agree’ (1) to ‘strongly disagree’ (6) and a total score is generated by summing the responses to all items. A cost-utility analysis using Quality Adjusted Life Years (QALY) was not attempted as this outcome measure is not suitable for this population [52, 53].

### Analyses of resource use, costs and outcome data

Resource use items were summarised by trial allocation group and follow-up period, and differences between groups were analysed using t-tests for continuous variables and chi-squared tests for categorical variables. Mean costs by cost category and mean total costs were estimated by trial allocation group for all time periods. Cost comparisons were carried out using Student’s t-tests. Differences in mean total costs and their CIs were estimated. Non-parametric bootstrap estimates based on 5,000 replications were also calculated for these differences in mean costs, and their respective CIs calculated.

A complete-case analysis approach was used whereby participants were included in the analysis regardless of attendance, excluding those lost to follow up (*n* = 27; 20 Intervention; 7 Comparison). Analyses were also conducted to identify any differences between those retained in the study and those who were lost to follow-up. At the outset of the trial, strategies were put in place to minimise missing cost data and a plan was developed to handle missing data to minimise potential biases. Between group differences at follow-up were assessed using Independent-samples t-tests on change scores calculated between baseline and 24 months. This approach was adopted in line with recommendations for analysis of change in non-randomised, naturalistic studies where equality at baseline is not presumed [54, 55].

### Cost-effectiveness analyses

Costs and outcomes were calculated over the trial time horizon (24 months) and discounted at 4% per annum as recommended by HIQA [33]. The cost-effectiveness results were primarily expressed in terms of an incremental cost-effectiveness ratio (ICER). This was calculated as the difference in mean costs divided by the difference in mean outcomes (PSOC score) between the PIN programme and SAU. The non-parametric bootstrapping approach was used to determine the level of sampling uncertainty surrounding the mean ICER by generating 5,000 estimates of incremental costs and benefits. These were represented graphically on four-quadrant cost-effectiveness planes. Cost-effectiveness acceptability curves (CEACs), showing the probability that the PIN programme is cost-effective relative to SAU across a range of cost-effectiveness thresholds, were also generated, based on the proportion of bootstrap replicates with positive incremental net benefits. Published estimates of willingness-to-pay for unit changes in the outcome measure are not publicly available. Consequently, statements about cost-effectiveness estimated on the basis of the PSOC measure, are based on a hypothetical range of values for the cost-effectiveness threshold ranging from €0–€1,000.

### Sensitivity analyses

Comprehensive probabilistic sensitivity analyses (PSA) were undertaken to examine the impact of parameter uncertainty on the outputs of the cost-effectiveness analysis. Further sensitivity analyses were performed to assess the impact of increasing the cost of the PIN intervention (to the level observed in other similar studies) and recalculating the cost-effectiveness excluding extreme cost outliers. All analyses were undertaken in Stata v17 as per the pre-specified health economics analysis plan and reported in line with The Consolidated Health Economic Evaluation Reporting Standards 2022 [56].

## Results

A total of 163 parent-infant dyads were incorporated into the economic analysis (86 in the PIN group and 77 in the SAU group). Participants were all mothers (mean age = 32 years; SD = 4.9) and almost 20% were lone parents (Table 2). Characteristics were comparable between arms except with respect to parity and infant age. Mothers in the intervention group were more likely to be primiparous, while infants in the SAU group were slightly older. Missing parent and infant baseline characteristics, resource use data and PSOC outcomes were low (0.009%). Income had a slightly elevated degree of missingness (0.02%). Participating parents attended, on average, 8.35 (SD = 5.2) programme sessions; 13% (14/106) did not attend any part of the PIN intervention. Given the low level of missing data (1.69% of data was missing for parents; 4.24% for infants), and profile of missingness (no more than one missing time period was observed for any individual out of the maximum four data collection time points), simple mean imputation (as opposed to multiple imputation) was employed. This approach was adopted to minimise bias potential [57].

**Table 2:** Participant Characteristics (figures are numbers (%) unless otherwise stated) adapted from Hickey et al., 2020 [28]

	**Intervention (PIN)** ***n* = 86**	**Comparison (SAU)** ***n* = 77**
**Lone parent**	18 (21)	14 (18)
**Mother mean age (SD)**	32.4 (4.9)	32.3 (5.1)
First time parent^*^	60 (71)	28 (36)
**Ethnic minority**	13 (15)	17 (22)
**Unemployed**	15 (18)	15 (19.5)
**Low income**^a^	26 (31)	21 (27)
**Male infant**	39 (46)	39 (51)
Infant mean age in months^*^ (SD)	1.8 (0.8)	2.06 (0.7)

^*^Significant differences between intervention and comparison group assessed using Chi-Square and Independent Samples t-tests; *p* < 0.05

^a^Based on 60% of the National Median Income (an equivalised disposable income per individual of 228.13/week; CSO, 2016)

### Programme costs

The total cost of delivering the PIN intervention amounted to €55,611. This comprises both capital and recurrent outlays. Capital costs are the one-time expenses that are required to set up and run the programme and may include the purchase of equipment, furniture, materials, curriculum, training, and evaluation tools. Recurrent costs are the ongoing expenses that are needed to maintain and operate the programme, such as staff salaries, rent, utilities, transportation, supplies, and maintenance. Staff costs constituted the largest cost component (accounting for 91%), with training costs, venue hire, catering, mileage accounting for the remainder. This represents a cost of €647 per parent-infant dyad on the basis of 86 parent-infant dyads having received the intervention. A high degree of variability was observed in training and group session costs between sites and providers. These were contingent on a variety of contextual factors, including number of participants per session, venue costs, training expenses and grade of staff undertaking training or delivery of the programme.

### Resource use and costs

Costs associated with resource utilisation for both parent and infants, are presented for baseline and subsequent follow-up time points (Table 3). There were no statistically significant between-group differences over the entire follow-up period, with the exception of the cost of outpatient appointments for parents and casualty attendance and ambulance costs for infants (Table 4). During this period, mean (SE) costs from an Irish health and social care perspective, inclusive of the cost of the programme, were €7,027 (SE €1,345) in the intervention arm and €4,811 (SE €593) in the control arm, generating a (non-significant) mean cost difference of €2,216 (bootstrap 95% CI -€665 to €5,096; *p* = 0.14).

**Table 3:** Resource use by group allocation, study period and resource allocation

	Baseline		Follow-up 1		Follow-up 2		Follow-up 3	
	Intervention	Comparison	Intervention	Comparison	Intervention	Comparison	Intervention	Comparison
**Parent**								
**GP visit**	1.49 (0.2)	1.1 (0.12)	1 (0.18)	1.29 (0.28)	1.92 (0.26)	1.41 (0.26	2.15 (0.33)	1.57 (0.26)
**Nurse visit**	1.27 (0.26)	0.84 (0.15)	0.21 (0.1)	0.18 (0.08)	0.1 (0.04)	0.03 (0.02)	0.07 (0.04)	0.08 (0.05)
**Health visitor**	0.21 (0.1)	0.05 (0.04)	0 (0)	0 (0)	0 (0)	0 (0)	0 (0)	0 (0)
**Social worker**	0 (0)	0 (0)	0.01 (0.01)	0.01 (0.01)	08 (0.54)	0 (0)	0.03 (0.02)	0 (0)
**Psychiatric nurse**	0 (0)	0 (0)	0 (0)	0.03 (0.03)	0 (0)	0 (0)	0 (0)	0 (0)
**Counsellor**	0.02 (0.02)	0.01 (0.01)	0.21 (0.15)	0.17 (0.10)	0.86 (0.56)	0.32 (0.21)	0.98 (0.66)	0.82 (0.48)
**Casualty**	0.08 (0.05)	0.01 (0.01)	0.04 (0.02)	0.01 (0.01)	0.08 (0.05)	0.05 (0.04)	0.03 (0.02)	0 (0)
**Outpatient**	0.21 (0.08)	0.13 (0.04)	0.26 (0.14)	0.1 (0.06	0.61 (0.17)	0.19 (0.08)	1 (0.4)	0.66 (0.21)
**Hospital stay**	0.06 (0.05)	0.04 (0.03)	0.77 (0.75)	0 (0)	1.29 (1.02)	0.38 (0.34)	0.43 (0.16)	0.19 (0.08)
**Specialist**	0.49 (0.12)	0.17 (0.05)	0.14 (0.07)	0.12 (0.08)	0.68 (0.42)	0.31 (0.16)	0.5 (0.13)	0.49 (0.21)
**Other**	0.36 (0.15)	0.12 (0.05)	0.41 (0.25)	0 (0)	0.23 (0.14)	0.14 (0.08	0.26 (0.12)	0.26 (0.13)
**Infant**								
**GP**	1.91 (0.12)	2.34 (0.16)	1.84 (0.15)	2.51 (0.3)	2.72 (0.33)	2.03 (0.29)	1.32 (0.2)	1.28 (0.16)
**Nurse**	2.74 (0.17)	2.6 (0.2)	1.55 (0.13)	1.84 (0.17)	1.13 (0.12)	0.89 (0.12)	0.94 (0.09)	0.62 (0.07)
**Health visitor**	0.31 (0.17)	0.05 (0.01)	0.01 (0.01)	0.47 (0.28)	0 (0)	0.08 (0.06)	0 (0)	0.01 (0.01)
**Social worker**	0.24 (0.19)	0 (0)	1.88 (1.66)	0 (0)	0.14 (0.14)	0.01 (0.01)	0.07 (0.07)	0 (0)
**Paediatrician**	0.22 (0.07)	0.08 (0.04)	0.07 (0.04)	0.09 (0.05)	0.04 (0.03)	0.03 (0.03)	0.02 (0.02)	0 (0)
**Casualty**	0.07 (0.03)	0.16 (0.08)	0.24 (0.06)	0.25 (0.06)	0.42 (0.08)	0.27 (0.07)	0.06 (0.03)	0.37 (0.14)
**Outpatient**	0.55 (0.09)	0.88 (0.25)	0.53 (0.12)	0.58 (0.2)	0.43 (0.11)	0.35 (0.12)	0.2 (0.06)	0.22 (0.08)
**Hospital stay**	0.27 (0.12)	0.34 (0.16)	0.12 (0.06)	0.66 (0.21)	0.39 (0.26)	0.33 (0.14)	0.15 (0.12)	0.13 (0.07)
**Ambulance**	0 (0)	0.01 (0.01)	0.04 (0.02)	0 (0)	4.4 (2.16)	0 (0)	0 (0)	0.01 (0.01)
**Specialist**	0.17 (0.08)	0.04 (0.02)	0.11 (0.04)	0.19 (0.09)	0.79 (0.76)	0.07 (0.04)	0 (0)	0 (0)
**Other**	0.48 (0.14)	0.27 (0.11)	0.19 (0.08)	0.19 (0.16)	0.04 (0.03)	0.03 (0.03)	0.11 (0.07)	0.01 (0.01)

**Table 4:** Cost-effectiveness acceptability curve

	**Group allocation, mean (SE) cost (€)**				
**Cost category by period**	**Intervention**	**Control**	**mean difference**	***p*-value**	**95% CI (€)**
**Parent**					
GP visit	344.10 (31.90)	275.98 (34.79)	68.12	0.15	-24.96 to 161.20
Nurse visit	53.27 (9.52)	38.06 (6.24)	15.21	0.19	-7.87 to 38.28
Health visitor	9.59 (4.42)	2.39 (1.68)	7.20	0.15	-2.57 to 16.97
Social worker	31.12 (21.24)	0.51 (0.51)	30.60	0.18	-13.86 to 75.06
Psychiatric nurse	0 (0)	0.85 (0.85)	-0.85	0.29	-2.46 to 0.75
Counsellor	129.16 (68.95)	83.13 (35.44)	46.02	0.57	-112.68 to 204.73
Casualty	69.88 (22.56)	23.51 (13.39)	46.37	0.09	-7.04 to 99.77
Outpatient	364.66 (79.51)	177.93 (42.45)	186.73	0.05	2.46 to 371.00
Hospital stay	1442.01 (745.20)	565.23 (327.12)	876.78	0.30	-776.49 to 2530.05
Specialist	309.04 (94.55)	191.80 (54.86)	117.24	0.30	-105.51 to 339.99
Other	220.45 (63.45)	90.12 (27.38)	130.32	0.07	-11.94 to 272.58
**Infant**					
GP visit	407.36 (27.93)	427.09 (32.94)	-19.73	0.65	-104.60 to 65.14
Nurse visit	207.96 (8.68)	198.71 (9.95)	9.25	0.48	-16.74 to 35.24
Health visitor	14.74 (8.03)	28.08 (13.30)	-13.34	0.38	-43.45 to 16.77
Social worker	26.73 (25.34)	0.51 (0.51)	26.21	0.32	-26.19 to 78.62
Paediatrician	13.36 (3.26)	6.82 (2.47)	6.54	0.12	-1.66 to 14.73
Specialist	192.11 (135.60)	67.93 (22.06)	124.18	0.39	-161.13 to 409.49
Casualty	657.09 (86.12)	986.00 (181.35)	-328.91	0.09	-711.57 to 53.75
Outpatient	303.53 (48.10)	366.43 (81.48)	-62.90	0.49	-244.55 to 118.74
Hospital stay	732.10 (278.98)	1338.30 (299.61)	-1,414.00	0.14	-1414.00 to 201.60
Ambulance	7.81 (3.62)	0 (0)	7.81	0.05	0.16 to 15.45
Other	122.44 (29.62)	92.63 (39.14)	29.82	0.54	-65.94 to 125.58

### Cost-effectiveness of the PIN intervention

**Fig. 3:**
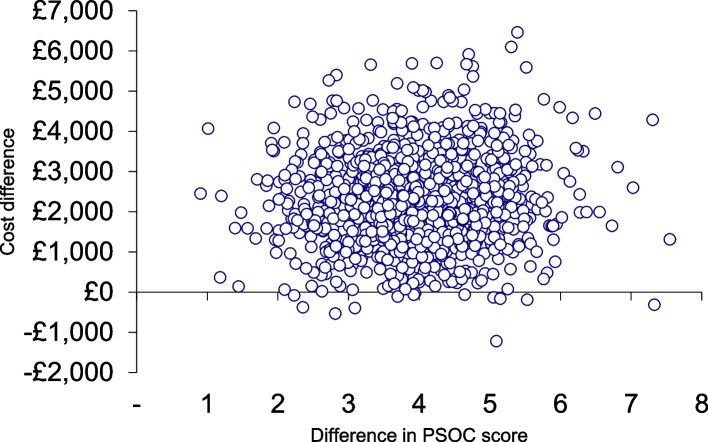
Cost-effectiveness plane

**Fig. 4:**
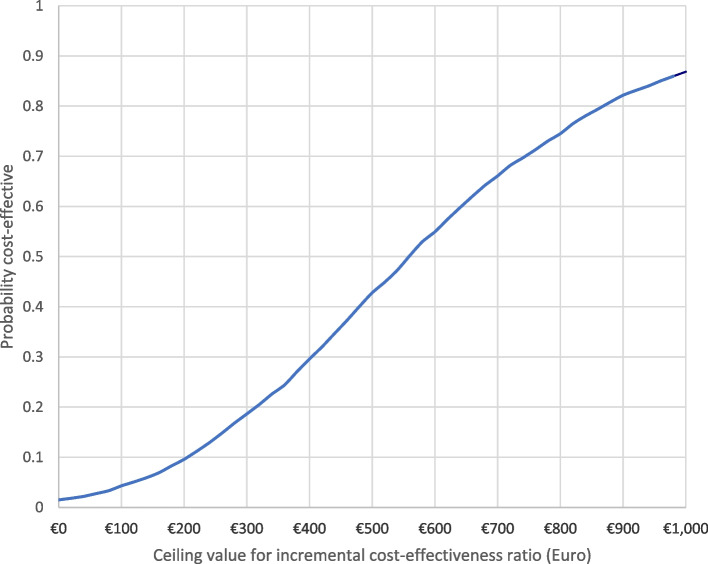
Group allocation, mean (SE) cost (€)

Statistically significant differences were found between the intervention and comparison groups on the primary outcome (PSOC total score); a mean (SE) PSOC total score of 0.71 (0.93) in the intervention arm and -3.20 (0.85) in the comparison arm, generated a mean difference of 3.91 (bootstrap 95% CI 1.44 to 6.38). The incremental cost-effectiveness of the PIN programme (see Table 5) was estimated at €614 per PSOC unit gained (bootstrap 95% CI €54 to €1,481). The intervention was associated with both a net positive cost and net positive effect; hence, the bootstrapped mean incremental cost effectiveness ratios (ICERs) fell largely in the north-east quadrant (Fig. 3)*.* The cost-effectiveness acceptability curve (CEAC) (Fig. 4) indicates that, at a willingness-to-pay of €1,000 per one unit change in PSOC, the probability that the PIN programme was cost-effective was 87%.

**Table 5:** Sample statistics and incremental cost-effectiveness results

	Treatment group (*n* = 86)	Control group (*n* = 77)	Difference	95% confidence interval
**Effect (PSOC)**				
Mean	0.71	-3.20	3.91	1.44 to 6.38
SE of mean	0.93	0.85		
**Cost**				
Mean	**€**7,027	**€**4,811	**€**2,216	-**€**665 to **€**5096
SE of mean	**€**1,345	**€**593		
**Cost and effect**				
Covariance	2,080	3,841	79	
Correlation	0.02	0.11	0.04

### Sensitivity and subgroup analyses

One-way deterministic sensitivity analysis, exploring the impact on cost-effectiveness of, for example, poor attendance at PIN sessions or increased cost of venues or category of staff, were not undertaken separately as such analyses do not take account of any correlations and non-linearities in the model. Other studies have reported higher parenting programme delivery costs; therefore, we explored the impact on cost-effectiveness associated with a doubling of delivery costs (from €647 to €1,294). This arbitrary assumption resulted in a mean incremental cost-effectiveness of €775 per PSOC unit gained (bootstrap 95% CI €188 to €1,735) and a 77% probability that the PIN programme was cost-effective at a willingness to pay of €1,000 per one unit change in PSOC. In the treatment group, two extreme outliers were observed (greater than €80,000) associated with extended hospital stays. An arbitrary capping of these values at €40,000 resulted in an incremental cost-effectiveness of €343 per PSOC unit gained (bootstrap 95% CI -€38 to €867) and a 99% probability that the PIN programme was cost-effective at a willingness to pay of €1,000 per one unit change in PSOC.

## Discussion

Our findings suggest that the PIN programme was cost-effective at a relatively low willingness-to-pay threshold when compared to SAU and using within trial data collected in a real-world setting; this cost-effectiveness remained unchanged when subjected to sensitivity analysis. The results show significant differences between parents who received the PIN programme and those who received SAU, suggesting that the programme led to positive outcomes in terms of parenting attitudes; conversely, those who received SAU experienced a diminished sense of competency over time. Recent research has suggested that parenting self-efficacy declines as children become older [58], although a more mixed picture in this regard, is reported in previous work [59]. Here, there were no significant cost differences observed between groups over time. Furthermore, at a willingness-to-pay of €1,000 per one unit change in the PSOC, the probability that the PIN programme was cost-effective, was high. Overall, parenting sense of competence is a frequently targeted change mechanism in early parenting interventions, but there is only a limited understanding, to date, of how parenting self-efficacy evolves as children grow and parents adapt to new parenting tasks. Further longitudinal studies are needed to assess the association between child age and parenting self-efficacy, as well as its role in the longer-term outcomes of early parenting interventions [44].

When interpreting the findings, the challenges involved in assessing the cost-effectiveness of preventative interventions in this population should be kept in mind [15, 29]. Although QALY is a widely used outcome measure for assessing the cost-effectiveness of health interventions, it was not used here as it assumes that health-related quality of life can be measured and valued on a single scale ranging from 0 (death) to 1 (full health). However, this may not capture the complexity and diversity of children's health and well-being, which may depend on factors such as developmental stage, cognitive abilities, social relationships, and environmental context. Moreover, the QALY may not reflect the preferences and values of children and their families, who may have different perspectives on what constitutes a good outcome. Instruments used to measure health-related quality of life may also not be valid or reliable for children, especially for very young or pre-verbal children who cannot self-report their health status. Furthermore, these instruments may not be sensitive to the specific attributes and domains that are relevant for children's health and well-being, such as growth, development, learning, play, and participation [53].

The well-established cost-utility framework used to assess clinical interventions is also less successful when applied to preventative interventions where costs and outcomes can fall across multiple sectors and intervention benefits may extend well beyond the time horizon of the study. Moreover, other less tangible benefits of early intervention, such as early identification of difficulties, or signposting of families to additional services and supports, are not explicitly quantified and valued within the current evaluative framework. Additionally, no explicit societal willingness-to-pay thresholds exist for outcomes commonly used to assess early years interventions, leading to greater difficulty in judging and comparing programmes. One possible approach to overcoming these challenges and capturing the cost effectiveness of early years interventions that have outcomes across multiple sectors, is to use a combination of cost–consequences analysis and cost–benefit analysis. Cost–consequences analysis presents the costs and outcomes of an intervention without aggregating or valuing them. Cost–benefit analysis, on the other hand, attempts to assign monetary values to all the costs and benefits of an intervention, including non-health and community benefits. This combined approach could help decision-makers choose interventions that maximise health and social benefits given the resources available and ensure their fair distribution across the population.

Gardner and colleagues [60] reported costs ‘as provided’ for IY parenting interventions of between £1,496 and £1,792 (based on 5 randomised trials). The magnitude of costs in our study were significantly lower, but comparable to those of the E-SEE trial which explored the cost-effectiveness of a proportionate parenting programme and reported intervention costs of £458.50 per dyad, and an ICER of £26,312/QALY [29]. The lower costs reported here, are likely due to the commissioning of components to voluntary sector organisations which were billed at a cost per component delivery irrespective of programme attendance, as well as the leveraging of resources available to participating organisations (e.g. health centres/community centres were used to deliver programmes to minimise venue hire costs where possible). Our results offer additional support to previous findings which suggest that course attendance significantly contributes to average programme costs [61] and that staff expenses constitute the largest cost component of delivering group sessions [13].

### Comparison to previous research and study strengths

A relatively small number of evaluations of parenting programmes have incorporated an assessment of costs and/or cost-effectiveness, most of which have been conducted with targeted programmes for parents of school-going children [62–65]. Ulfsdotter and colleagues [28] explored the effectiveness and cost-effectiveness of a universal group-based programme, but with parents of older children (3–12 years). A small number of other studies have focused on younger children (0–2 years) [19, 25, 27, 29, 66]. Thus, this study addresses an important knowledge gap and provides important practical information for policy makers who wish to commission services aimed at enhancing parental competency.

Recent studies highlight the challenge of determining the cost-effectiveness of early intervention programmes [28, 29]. Despite a growing commitment to children’s rights and the development of prevention-focused policies, the wellbeing of children and families have been negatively impacted by austerity, COVID-19 and cost of living increases [67–69]. Strengthening social, emotional and mental health functioning requires complex, interagency approaches, but these kinds of interventions can be difficult to cost within the traditional cost-effectiveness framework. Recent calls have been made to move beyond market-centred approaches towards more rights-based and creative, open-minded collaborations between health economists, researchers, service providers and policy makers [70]. Overall, there remains very little robust evidence on the cost-effectiveness of children’s services, while the appropriateness of traditional cost-effectiveness approaches in the context of complex early interventions in the primary health and social care sector, requires further consideration [71].

### Study limitations

Our study has a number of limitations. First, the primary outcome measure used in the cost-effectiveness analysis, was a measure of parental competence as opposed to a child development outcome. However, parents were the primary target of the intervention, while parental self-efficacy is an important targeted outcome of parenting interventions and is associated with a number of positive parent and child outcomes, including better parenting skills, as well as positive parent and child functioning [43, 59]. Second, economic evaluation of public health interventions is complex and presents a range of challenges for health economists. The outcome of choice for the economic evaluation of clinical interventions is typically the quality adjusted life year (QALY) where for example in the UK, a societal willingness-to-pay threshold of between £20,000-£30,000 per QALY gained is applied. No such threshold exists for the PSOC (or other measures frequently used in the assessment of early years interventions); hence funders must determine, within their current budget, whether a €1,000 per unit increase in PSOC is a worthy investment, and whether this investment should be targeted at those considered to have greater parenting risks.

Third, randomised controlled trials (RCTs) are considered to be the gold standard for causal inference, but under certain conditions, quasi-experimental designs that lack random assignment have also been shown to produce credible results [72]. Nevertheless, we cannot rule out that this design may have resulted in response bias, although an RCT was precluded by ethical and practical concerns expressed by participating organisations [31]. Fourth, our results are based on a limited costing perspective and relate only to the duration of the study which may underestimate societal impacts. Costs relating to the educational sector, justice and voluntary sectors are not included. If results are to be used for decision-making, the implications of such downstream costs should be considered. Fifth, the data here involved parent-only reports and relied on recall of service utilisation.

Sixth, engagement and retention were relatively low and study participants were all self-referred. There were more first-time parents in the intervention group; however, there were no differences in baseline PSOC scores between first time mothers and those with additional children, although previous literature has demonstrated inconsistent effects of parity on parental self-efficacy [73]. Despite these limitations, our study represents an important contribution to the little existing evidence and knowledge on the cost-effectiveness of universal parenting programmes delivered in real-life settings and especially in the crucial earliest years.

## Conclusion

This study provides evidence for the potential cost-effectiveness of a group-based early parenting intervention delivered on a universal basis in primary health care and community-based settings. The findings provide important information for practitioners and policy makers in this area**.** Overall, there remains very little evidence regarding the cost-effectiveness of early parenting interventions, and methodological limitations in this area remain a considerable challenge. Much more research is needed in this area, including economic evaluations, in order to enhance the implementation of high-quality programmes that best meet the needs of families and young infants, and to ensure that they offer the most efficient use of available resources. Despite the attractiveness of universal interventions as a means of reaching larger numbers of families and removing the stigma associated with targeted interventions, there remains limited evidence for the effectiveness and cost effectiveness of universal prevention as a public health instrument [74]. Thus, a need for further research and particularly large-scale high quality trials, is indicated. Further consideration of how best to assess the cost-effectiveness of preventative parenting interventions implemented in the earliest years, is also needed.

### Supplementary Information

**Additional file 1: Appendix 1.** Irish Health Information and Quality Authority (HIQA) resource valuation guidelines.

## Data Availability

The datasets generated and/or analysed during the current study are not publicly available due to lack of participant consent but are available from the corresponding author on reasonable request.
