# Supplementary material for: A cost-effectiveness analysis of a universal, preventative-focused, parent and infant programme
Source: BMC Health Serv Res. 2024 Feb 8;24:176. doi: 10.1186/s12913-023-10492-w (PMC10851506; doi:10.1186/s12913-023-10492-w)
Supplement: Supplementary file 1 — Additional file 1: Appendix 1. Irish Health Information and Quality Authority (HIQA) resource valuation guidelines. [file 12913_2023_10492_MOESM1_ESM.docx]

**Supplementary materials**

***Appendix 1: Irish Health Information and Quality Authority (HIQA) resource valuation guidelines***

Following HIQA guidance^1^, the mid-point of the appropriate grade of staff was identified using the consolidated salary scales available from the national health service in Ireland, the Health Service Executive (HSE). HSE associated non-pay costs (for nurses, psychiatric nurse, health visitor and social worker) were estimated in accordance with Regulatory Impact Analysis (RIA) guidelines issued by the Department of the Taoiseach. This method included adjustments for non-pay costs associated with hiring additional staff, including employers’ PRSI, superannuation, as well as general overheads such as rent, light and heat, office facilities, telephone, general supplies etc. Twenty-five percent of direct salary cost was applied, with net pension cost as a percentage of pensionable remuneration estimated at 4% for healthcare workers in the public sector^2^. The cost of mediation and counselling was based on a per hour estimate from the Mediators Institute of Ireland, and average clinical psychologist pay scale respectively, to which Employers PRSI, pension and overhead costs were applied.

The cost of a GP consultation was based on published data, with the estimate comprising a mix of capitation, fees and allowances, plus payments under the Maternity and infant care scheme and for non-card-holders^3^. Costs for an A&E visit, outpatient consultation and in-patient stay (per night) were supplied by the Healthcare Pricing Office (HPO) for 2018^4^. These figures were based on average cost across all hospitals and specialities. The cost of in-patient stay included treatment, care and hotel costs (but excluded capital and depreciation). The cost of an ambulance journey and community paediatrician was based on previously published estimates updated to 2018 prices.

1 https://www.hiqa.ie/sites/default/files/2020-09/HTA-Economic-Guidelines-2020.pdf. Accessed 20^th^ December 2023

2 [https://www.hse.ie/eng/staff/resources/hr-circulars/final-1-march-2023-salary-scales.pdf Accessed 20th December 2023](https://www.hse.ie/eng/staff/resources/hr-circulars/final-1-march-2023-salary-scales.pdf%20Accessed%2020th%20December%202023)

3 Smith S, Jiang J, Normand C, O'Neill C. Unit costs for non-acute care in Ireland 2016-2019. HRB Open Res. 2021 Apr 23;4:39. doi: 10.12688/hrbopenres.13256.1. PMID: 35317302; PMCID: PMC8917322.

4 <https://hpo.ie/> Accessed 20 December 2023

5 O'Neill D, McGilloway S, Donnelly M, Bywater T, Kelly P. A cost-effectiveness analysis of the Incredible Years parenting programme in reducing childhood health inequalities. Eur J Health Econ. 2013 Feb;14(1):85-94. doi: 10.1007/s10198-011-0342-y. Epub 2011 Aug 19. PMID: 21853340.
